# Developing a new electromyography-based algorithm to diagnose the etiology of fecal incontinence

**DOI:** 10.1007/s00384-014-1859-1

**Published:** 2014-04-18

**Authors:** Michał Nowakowski, Krzysztof A. Tomaszewski, Roman M. Herman, Jerzy Sałówka, Michał Romaniszyn, Mateusz Rubinkiewicz, Jerzy A. Walocha

**Affiliations:** 1 Department of Medical Education, Jagiellonian University Medical College, Krakow, Poland; 2 Department of Anatomy, Jagiellonian University Medical College, Krakow, Poland; 3 Department of Clinical and Experimental Surgery, Jagiellonian University Medical College, Krakow, Poland; 4 Department of General Surgery, Stanley Dudrick Memorial Hospital, Skawina, Poland; 5 Department of General Surgery, G. Narutowicz Specialist City Hospital, Krakow, Poland

**Keywords:** Computer-assisted diagnosis, External anal sphincter, Fecal incontinence, Neurogenic, sEMG

## Abstract

**Purpose:**

For surface electromyography (sEMG) to become widely used in fecal incontinence (FI) etiology assessment, one would have to create a simple, step-by-step, computer-aided, electromyography-based algorithm that would become the basis for a computer-aided diagnosis (CAD) system. Thus, the aim of this work was to develop such an algorithm.

**Methods:**

Each patient included in the study underwent a structured medical interview, a general physical examination, and a proctological examination. Patients that scored more than 10 points on the fecal incontinence severity index (FISI) underwent further tests that included rectoscopy, anorectal manometry, transanal ultrasonography, multichannel sEMG, and assessment of anal reflexes. Patients with fully diagnosed FI were included into the study group. The control group consisted of healthy volunteers that scored five or less points on the FISI and had no known anal sphincters dysfunction.

**Results:**

Forty-nine patients were qualified to the study group (age ± SD 58.9 ± 13.8). The control group was number- and gender-matched (age ± SD 45.4 ± 15.1). The sensitivity and specificity of classification tree number I, to diagnose neurogenic FI, were 89.5 and 86 %, respectively. For patients with idiopathic FI, these values were 82 and 91 %, respectively. The sensitivity and specificity of classification tree number III, to diagnose neurogenic FI, were 84 and 78 %, respectively. For patients with idiopathic FI, these values were 78 and 87 %, respectively.

**Conclusions:**

The relative simplicity and low classification costs allow to assume that algorithms based on classification trees I and III will serve to be the basis for a FI etiology CAD system.

## Introduction

The International Continence Society (ICS) has defined fecal incontinence (FI) as the involuntary loss of liquid or stool that is a social or hygiene problem [1]. The prevalence of FI in adults varies between reports from 0.5 to 18 % [2–4]. FI becomes more common with age, with approximately one third of people over 65 years of age having symptoms at least once a year [1, 5]. FI prevalence, apart from being age related, is also gender dependent, with over 60 % of affected elderly being women [1]. However, this last statement is not necessarily true for every population [6]. The many FI causes include trauma (obstetric, iatrogenic), radiation damage, rectal prolapse, inflammatory bowel diseases, neurological disorders, and cognitive impairment [1, 3]. FI etiology assessment can be divided into physiological and structural. Methods used for the physiological assessment of FI cause include anorectal manometry, needle and surface electromyography (sEMG), and pudendal nerve terminal latency [1]. Clinical usefulness of the last-mentioned test for innervation assessment has been questioned over the last years by many authors [7]. External anal sphincter (EAS) structural assessment includes ultrasonography and MRI [1]. It is crucial for effective treatment to have clear understanding of the etiology of FI with different options offered to patients with neurogenic, myogenic, or idiopathic FI. Although sEMG has been superseded by transanal ultrasound in identifying localized EAS injuries, there are some reports showing that it can be useful in determining the etiology of FI [8]. Unfortunately, interpreting EAS electromyograms is difficult, as there is little data to compare the obtained results to [9–11]. Thus, up to date, no further attempts have been made to use sEMG in determining the etiology of FI. For sEMG to become widely used in FI etiology assessment, one would have to create a simple, step-by-step algorithm that would be based on objective numerical values, rather than on subjective graphic electromyogram interpretation. Up-to-date literature lacks such algorithms. These could be used in the future to create an FI etiology computer-aided diagnosis (CAD) system.

Thus the aim of this work was to develop a simple, computer-aided, electromyography-based algorithm to diagnose the etiology of FI.

## Patients and methods

### Patients

Patients were recruited for this study from among the ones referred to the outpatient clinic of the Third Department of General Surgery (Jagiellonian University Medical College, Krakow, Poland) with diagnosis of fecal incontinence. Each patient included in the study underwent a structured medical interview, a general physical examination, and a proctological examination. In addition, she or he filled out the fecal incontinence severity index questionnaire (FISI) [12].

### Study group

Patients that scored more than 10 points on the FISI underwent further tests that included rectoscopy, anorectal manometry, transanal ultrasonography, multichannel sEMG, and assessment of anal reflexes. Based on the outcomes of the abovementioned tests, FI was diagnosed and its etiology established. After best available assessment of FI etiology, patients were included into the study.

All patients were subdivided by FI etiology into three groups. The myogenic FI group included patients with anal sphincter injury or atrophy confirmed on transanal ultrasound. Neurogenic FI was considered when patients displayed deficits on neurological examination (e.g., lack of perineal reflexes, lack of urge to defecate, or sensory deficits in sacral (S2–S4) spinal nerve innervation zones). Finally, idiopathic FI was considered when there were no abnormalities in the innervation or structure of the anal sphincters. Study group exclusion criteria were the following: age below 18 or above 90; pregnancy; lack of consent to participate in the study; not being able to participate in all the necessary test from the study protocol; and conditions that influenced the FISI score (other than fecal incontinence, including but not limited to diarrhea and inflammatory bowel disease).

### Control group

The control group consisted of healthy volunteers wishing to participate in the study. Each person from the control group underwent the same tests as patients from the study group. To be included in the control group, one had to score five or less points on the FISI and have no EAS dysfunction (assessed by transanal ultrasonography and anorectal monometry). Additional control group inclusion criteria included the following: age between 18 and 85; no anorectal complaints, no history of anorectal surgery; no history of diseases that might impair EAS innervation like diabetes, neuropthies, central nervous system disorders, and systemic disorders; for women—no labor-induced perineum injuries and not more than one pregnancy; and no abnormal findings on the performed tests.

### Tests

sEMG was performed using a prototype EMG-16 multichannel signal enhancer (OT Bioelettronica, Turin, Italy). The frequency used was between 10 to 500 Hz (3 dB). Sampling was set at 2,048 samples per second. The transducer used was a NI DAQ_MIO16 E-10 (National Instruments, USA) and attached to a standard PC. Measurements were made using an anorectal probe (diameter 14 mm) with three rings of 16 silver bar electrodes each. Each electrode was 1 × 9 mm large, and the distance between each ring was 8 mm. The measurements were made on the following depths, counting from the internal rim of the anus—from the anal pecten to 9 mm deep; from 18 to 27 mm deep; from 35 to 44 mm deep. After inserting the probe and waiting for 1 min for the patient to relax, three 10-s recordings were performed (one on each depth), with the patients’ EAS relaxed. Next, the same measurements were repeated with the EAS contracted. The whole procedure was repeated two times. Signal analysis was performed using Matlab. To analyze the amplitude root-mean-square (RMS) was calculated, according to the following equation:

$$ \mathrm{Xrms}=\sqrt{\frac{a_1^2+{a}_2^2+\cdots +{a}_n^2}{n}}. $$

RMS was calculated for each of the 16 electrode pairs, in each of the three rings, both during relaxation and contraction of the anal sphincter. Next, mean RMS was calculated for each of the three depths. Median frequency (MF) was calculated similarly to RMS [8].

Anorectal manometry was performed according to the minimum standards for anorectal manometry [13] of the European Neurogastroenterology and Motility Association. The test was performed using perfusion manometry (Polygraf HR Uro, Synectics Medical, Sweden) with a three-channel manometric probe. Maximal basal pressure (MBP) was defined as the maximal pressure during rest in any part of the anal canal and was the mean of three measurements. Maximal squeeze pressure (MSP) was defined as the maximal pressure during voluntary sphincter contraction in the same localization where MBP was previously measured. Transanal ultrasonography was performed using a Logiq 7 ultrasound machine (General Electric, Great Britain), with a 10–16 MHz probe [14].

### Statistical analysis

Statistical analysis was performed using the Statistica 10 software (StatSoft, Tulsa, USA). Elements of descriptive statistics were used—mean, standard deviation, and percentage distribution. To assess differences between groups, the Student’s *t* test was used. Classification and regression tree models were used to create an automatic decision algorithm to diagnose the etiology of FI. This model assigns patients to one of the groups (control vs. myogenic FI vs. neurogenic FI vs. idiopathic FI) based on known factors (RMS and MF). Results were presented as classification trees, which are used to predict membership of cases or objects in the classes of a categorical dependent variable from their measurements on one or more predictor variables. *V*-fold cross-validation was used to optimize and simplify the decision trees. In this study, the *V* value was set at 10. The significance level was set at *p* < 0.05.

### Ethics

All patients gave their informed consent prior to inclusion into the study. The research protocol was approved by the Jagiellonian University Ethics Committee. The study has been performed in accordance with the ethical standards laid down in the 1964 Declaration of Helsinki and its later amendments.

## Results

Overall, 55 patients were qualified to the study group, but due to artifacts in the sEMG recording, only 49–39 women (79.6 %) and 10 men were finally included in the analysis. sEMG recordings of the six excluded patients contained too many artifacts that resulted mainly from the loss of contact of several of the 36 recording electrodes or significant cross-talk from other muscles due to movement. In two of the six mentioned cases, there was also a significant 50 Hz interference. In all six cases, automated analysis of signal was considered unreliable. Two hundred and thirty-seven (179 women and 58 men) patients were qualified to the control group. From this group, 39 women and 10 men were chosen to be gender- and age-matched with the study group. The mean age (±SD) of the study group was 58.9 ± 13.8 and of the control group 45.4 ± 15.1. Table 1 presents the mean age of different subgroups of patients. The paired *t* test showed no significant differences in the mean age of men from the study and the control group (*p* = 0.74). Women from the study and control groups differed in mean age (*p* < 0.001). Taking into account the FISI score, the study group differed significantly from the control group (41.8 ± 11.4 vs. 4.1 ± 0.3, respectively; *p* < 0.0001). The study group was divided according to the diagnosed etiology of FI into myogenic FI (*n* = 6), neurogenic (*n* = 19), and idiopathic (*n* = 24). Statistically significant differences in the sEMG parameters between the abovementioned groups and the control group are presented in Table 2.

**Table 1:** Mean ages of different subgroups of patients

Patient subgroup	Mean age (women)	Mean age (men)	*p* value
Whole group	51.5	54.9	0.390
Study group	60.2	53.8	0.193
Control group	42.7	55.9	0.012

**Table 2:** Statistically significant differences in sEMG parameters between groups of patients with different FI etiology and the control group

	Neurogenic FI *n* = 19	Myogenic FI *n* = 6	Idiopathic FI *n* = 24	Control Group *n* = 49	*p* value
RMS 1C	0.0118			0.02246	0.006
RMS 3C	0.0069			0.01401	0.000
RMS 1R			0.01520	0.0093	0.008
RMS 2R			0.00853	0.0059	0.005
MF 1R		116.5078		97.265	0.008
MF 2R		115.1845		95.987	0.006
MF 3R		120.2160		93.915	0.006
MF 3R	106.8772			93.91531	0.041

Results presented as means. Numbers after RMS or MF refer to the depth of the ring that was used to obtain the measurements (1 closest to the anus; 3 the deepest ring)

*FI* fecal incontinence, *RMS* root-mean-square, *MF* median frequency, *R* external anal sphincter relaxed, *C* external anal sphincter contracted

### Classification trees

The below-described classification trees use the mean RMS and MF values (registered with the EAS relaxed/contracted and on different depths) to distinguish between different patient groups. The grouping variable for all the below-mentioned trees is FI etiology.

**Fig. 1:**
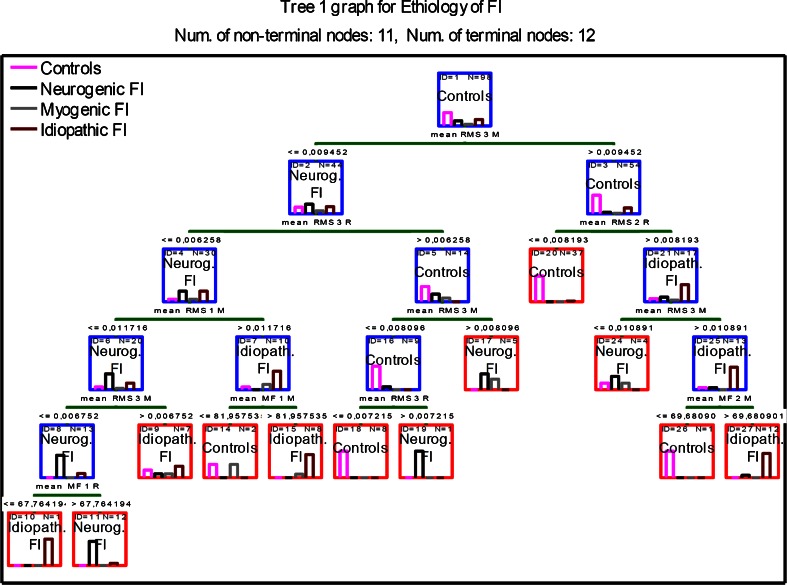
Classification tree number I (11 decision (nonterminal, *blue boxes*) nodes and 12 endpoints (terminal nodes, *red boxes*)) fecal incontinence etiology. Tree optimized taking into account minimal classification costs. *Bars inside each box* depict classified patients, divided by etiology (controls, neurogenic, myogenic, and idiopathic fecal incontinence). *Green bars* depict decisions with the numerical value below and the name of the variable above. Results presented as means. *FI* fecal incontinence, *RMS* root-means-square, *MF* median frequency, *R* external anal sphincter relaxed, *C* external anal sphincter maximally contracted. *Numbers* after RMS or MF refer to the depth of the ring that was used to obtain the measurements (*1* closest to the anus; *3* the deepest ring)

Figure 1 presents the classification tree number I. This tree correctly classified 46 (93.8 %) cases from the control group. One was incorrectly classified as having neurogenic FI and two as having idiopathic FI. Out of the neurogenic FI subgroup, 17 (89.5 %) cases were classified correctly and two incorrectly as having idiopathic FI. Among the 24 patients with idiopathic FI, 22 (91.7 %) were classified correctly and two incorrectly—one to the neurogenic FI subgroup and one to the control group. This type of tree was unable to correctly classify cases with myogenic FI—all of the six patients were incorrectly classified. The sensitivity and specificity of tree number I to diagnose neurogenic FI were 89.5 and 86 %, respectively. For patients with idiopathic FI, these values were 82 and 91 %, respectively.

**Fig. 2:**
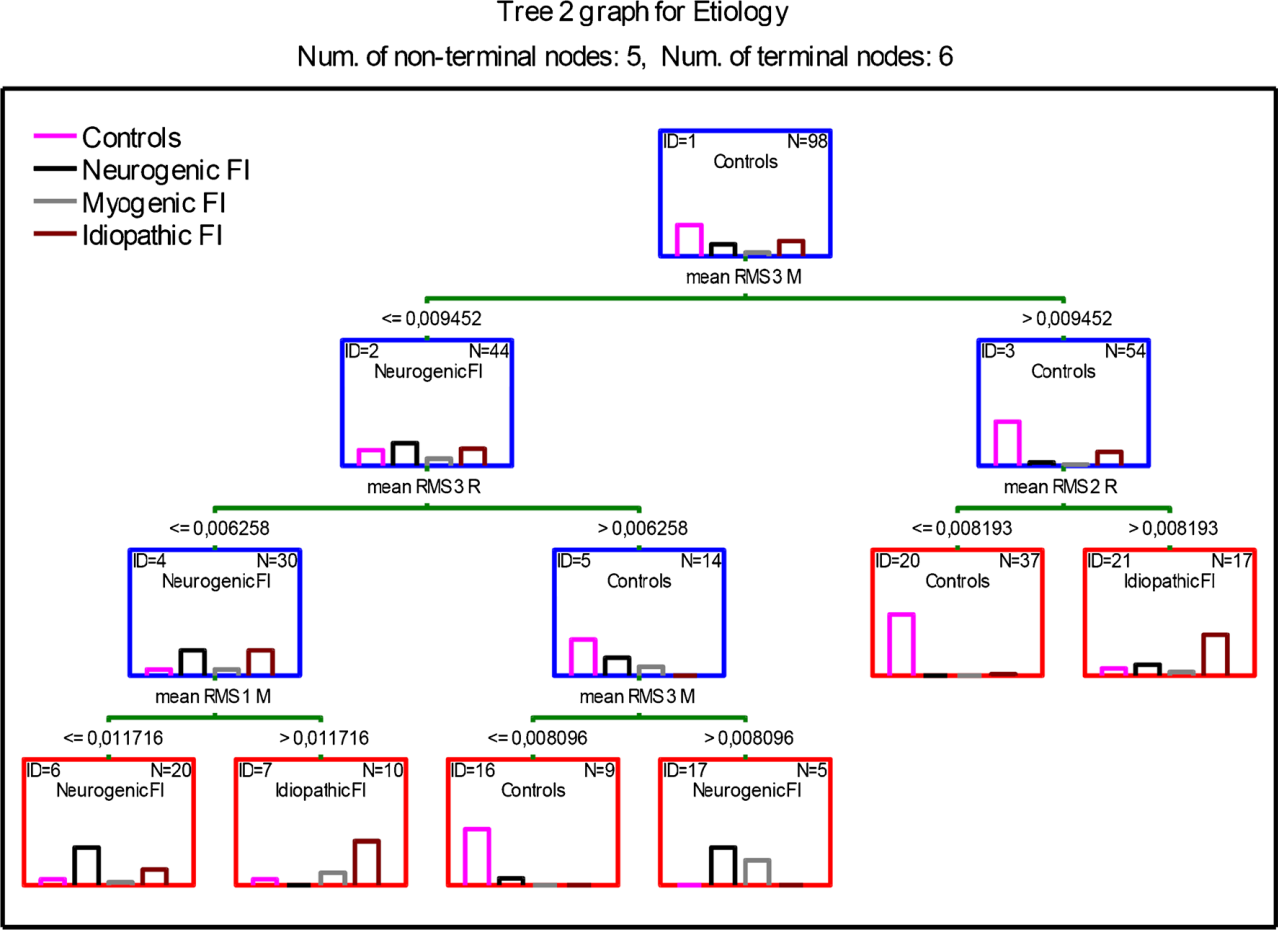
Classification tree number II (five decision (nonterminal, *blue boxes*) nodes and six endpoints (terminal nodes, *red boxes*)) fecal incontinence etiology. Tree optimized taking into account the minimal number of decision nodes. *Bars inside each box* depict classified patients, divided by etiology (controls, neurogenic, myogenic, and idiopathic fecal incontinence). *Green bars* depict decisions with the numerical value below and the name of the variable above. Results presented as means. *FI* fecal incontinence, *RMS* root-mean-square, *MF* median frequency, *R* external anal sphincter relaxed, *C* external anal sphincter maximally contracted. *Numbers* after RMS or MF refer to the depth of the ring that was used to obtain the measurements (*1* closest to the anus; *3* the deepest ring)

Due to the complexity (11 decision nodes) of tree number I presented in Fig. 1, the authors have decided to explore the data further and look for a simpler one. The result of further analysis is presented in Fig. 2. The presented tree (number II) has only five decision nodes and six endpoints. This tree correctly classified 89.8 % of cases from the control group, 79 % of patients with neurogenic FI, 75 % of patients with idiopathic FI, and none having myogenic FI. In spite of its simplicity, this tree correctly classified only 77 out of 98 patients (78.6 %), which was deemed unsatisfactory and was the reason for discarding this tree.

**Fig. 3:**
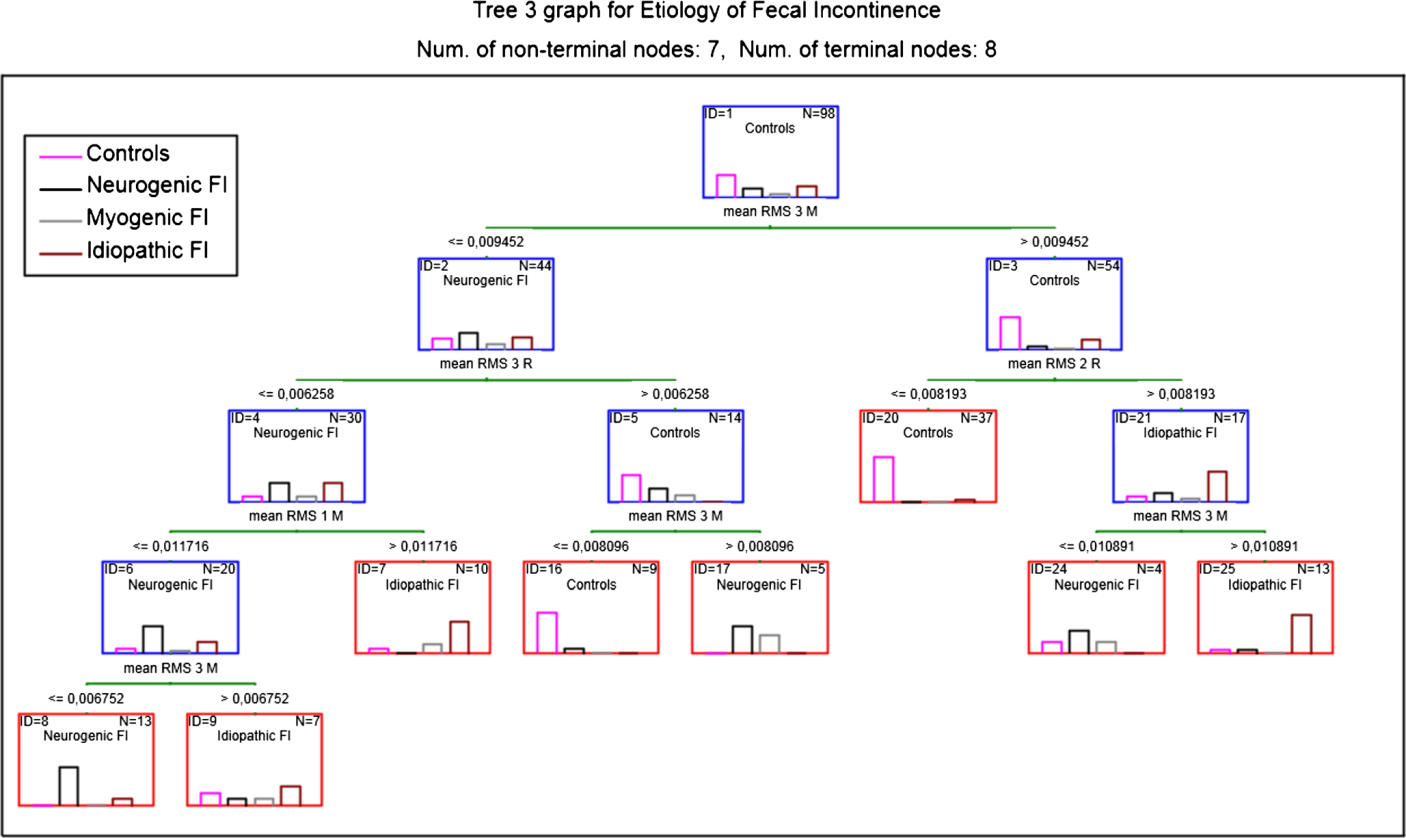
Classification tree number III (seven decision (nonterminal, *blue boxes*) nodes and eight endpoints (terminal nodes, *red boxes*)) fecal incontinence etiology. Tree optimized taking into account classification costs and the number of decision nodes. *Bars inside each box* depict classified patients, divided by etiology (controls, neurogenic, myogenic, and idiopathic fecal incontinence). *Green bars* depict decisions with the numerical value below and the name of the variable above. Results presented as means. *FI* fecal incontinence, *RMS* root-mean-square, *MF* median frequency, *R* external anal sphincter relaxed, *C* external anal sphincter maximally contracted. *Numbers* after RMS or MF refer to the depth of the ring that was used to obtain the measurements (*1* closest to the anus; *3* the deepest ring)

Tree number III (Fig. 3), with its seven decision nodes and eight endpoints, seems as the most optimal choice. Overall, it correctly classified 82.7 % of patients. The sensitivity and specificity of this tree to diagnose neurogenic FI were 84 and 78 %, respectively. For patients with idiopathic FI, these values were 78 and 87 %, respectively. A more detailed classification description, regarding tree number III, is presented in Table 3.

**Table 3:** Classification results for tree number III

	Observed value	Foreseen value				Total
		Control group	Neurogenic FI	Myogenic FI	Idiopathic FI	
Number of cases	Control group	44	1		4	49
Percentage of cases		89.80 %	2.04 %	0.00 %	8.16 %	
Number of cases	Neurogenic FI	1	16		2	19
Percentage of cases		5.26 %	84.21 %	0.00 %	10.53 %	
Number of cases	Myogenic FI		3		3	6
Percentage of cases		0.00 %	50.00 %	0.00 %	50.00 %	
Number of cases	Idiopathic FI	1	2		21	24
Percentage of cases		4.17 %	8.33 %	0.00 %	87.50 %	

*FI* fecal incontinence

## Discussion

The aim of this study was to develop a simple, computer-aided, electromyography-based algorithm to diagnose the etiology of FI. The created algorithms could be used in the future to construct a CAD tool for FI etiology assessment.

The appropriate assessment of FI etiology is crucial for choosing the right type of treatment. With the sole exception of sphincter injury, which is easily enough diagnosed with the use of either transanal ultrasound or MRI, fecal incontinence etiology remains difficult to assess due to lack of a verified diagnostic tool [15]. In regular proctological patient assessment, the diagnosis of neurogenic FI is largely based on history and physical examination including neurological examination. Due to the subjective nature and significant difficulty of the examination, other methods of FI etiology differentiation have been sought. Over the last few years, sEMG has been found to be useful in the analysis of EAS innervation [16]. There are also some reports showing that sEMG can be helpful when trying to diagnose the underlying cause of FI [8]. However, up to date, there are no studies that fully show that EAS sEMG assessment is reliable. We also have to remember that multichannel sEMG, in the form used in this study (specially created for the needs of the European Commission OASIS program) [9, 16], offers multichannel surface, hence accurate and noninvasive innervation assessment.

The lack of studies similar to this one limits the possibilities of this discussion. There is only one work that uses a similar sEMG recording technique and assesses the distribution of functional potentials in the EAS [8]. However, it omits the analysis of potential frequency or amplitude and addresses only healthy individuals. It was the lack of studies similar to this one that persuaded us to use the nonstandard statistical methods, such as the classification and regression tree models.

The popularity of CAD has grown over the years. Nowadays, it is a very popular technique most often used in radiology [17]. However, studies developing CAD methods for use in coloproctology are scarce. A study by Tischendorf et al. [18] present and automated classification system of colonic polyps based on the narrow-band imaging vascularization features. However, the authors conclude that the developed method is feasible, but classification by observers is still superior. Another study by Gardiner et al. [19] focus on developing a CAD system for the diagnostics of FI, basing on the pudendal nerve latency test (PNTML) and anorectal monometry. However, the usefulness of this study might be questionable as the PNTML is a measure of latency alone and not innervation as such. That is why it might not be relied on in the FI diagnostic process.

Developing a CAD system, based on sEMG, for the assessment of FI etiology is essential, as interpreting sEMG results is difficult and requires time and experience. Even for sEMG experts, a sEMG recording that lasts about 20 s may take up to 30 min to review, and this does not include identification and counting of motor unit action potentials. This greatly limits the popularity of sEMG. To prepare a CAD system, for FI etiology diagnosis, first, a suitable algorithm must be developed. Our study focused on this part, using the widely accepted statistical method of classification and regression trees with *V*-fold cross-validation.

Classification tree number I was optimized taking into account minimal classification costs (number of incorrectly classified cases) and maximum effectiveness in assigning the selected subjects. Using this tree, the classification costs were very low (13 %). Even though this tree was not able to assign correctly any patients with myogenic FI, this was not considered to be a problem. Tree number I was not optimized to classify myogenic FI, as diagnosing this type of FI is fairly easy with the use of transanal ultrasound y. We have developed several other classification trees that better dealt with classifying myogenic FI, but they were discarded due to significantly higher complexity and classification costs. The sensitivity and specificity values of tree number I, to both diagnose idiopathic and neurogenic FI, make this algorithm acceptable for clinical use.

Due to the complexity of tree number I, the authors have decided to use the *V*-fold cross-validation to explore for other trees that could be either less complex or have lower classification costs. For *V*-fold cross-validation, the *V* value was set at 10 to create 10 trees with different characteristics. Next, the trees were reviewed based on the number of decision nodes, classification costs, and general complexity.

Classification tree number III seems as the optimal choice for creating a CAD system, with only seven decision nodes, eight endpoints, and a maximal route of four nodes, taking into account the complexity of the analyzed group (98 cases, 12 analyzed parameters). Classification correctness of about 83 % seems relatively low, but when confronted with the fact that no such algorithms exist, and that at the moment differentiating FI etiology is often not possible (if it is not related to sphincter injury), it can be recommended for clinical use. The total classification costs were 17 %, but 35 % of these costs were generated due to patients with myogenic FI. Once again, the authors did not decide to optimize the tree to diagnose myogenic FI to keep the tree as simple as possible. It was decided that in the case of myogenic FI patients, the diagnosis will be based on transanal ultrasound. Excluding myogenic FI patients from the algorithm would not only lower the classification costs to 11 %, which is an impressive result, but would also increase the sensitivity and specificity of the tree.

The most significant limitation of this study is the etiological FI classification of the included patients. We used our best judgment, clinical experience, as well as available diagnostic tests to diagnose the possible etiology of FI of the patients included in this study. However, it is still possible that other surgeons would classify those patients differently. Moreover, it frequently happens that patients have more than one possible FI cause. Especially in the EAS injury group, we observed a significant heterogeneity in EMG signals. The possible stochastic effect of the injury may or may not include the neuromuscular junction (innervation zone) of the EAS. Thus, the injury may lead to an isolated muscular injury or also include a neurogenic component. We do understand that there are many patients with polietiological FI; however, for the purpose of this study, if an innervation deficit was present, the patient was classified as having neurogenic FI. The rationale for this was that the main focus of this study was to develop an algorithm which would differentiate patients with neurogenic FI from patients with FI of other etiology.

Another possible limitation of this study is the fact that the age of women in the control group differs significantly from the mean age of the study group. This occurred due to the fact that the authors were unable to recruit, into the control group, enough healthy women over 50 years of age. However, after statistical consultation, it was agreed that this difference, taking into account the rest of the group parameters, is acceptable.

The most important thing that the authors would like to achieve through this study is to develop a more straightforward method of identifying patients who due to the neurogenic etiology of their disease are less likely to respond to biofeedback therapy and have worse prognosis with sphincter repairs.

## Conclusions

The relative simplicity and low classification costs allow to assume that algorithms based on classification trees I and III will serve to be the basis for a FI etiology CAD system. Using such a system will simplify the process of FI etiology diagnosis. Furthermore, not only physicians will be able to use it but also medical staff that does not have profound knowledge of electrophysiology and biological signal computer analysis.
